# Sclerosing Mediastinitis Causing Unilateral Pulmonary Edema Due to Left Atrial and Pulmonary Venous Compression. A Case Report and Literature Review

**DOI:** 10.21470/1678-9741-2018-0067

**Published:** 2019

**Authors:** Nikolaos Panagopoulos, Vasileios Leivaditis, Pantelis Kraniotis, Panagiota Ravazoula, Efstratios Koletsis, Dimitrios Dougenis

**Affiliations:** 1 Department of Cardiothoracic Surgery, University Hospital of Patras, Patras, Greece.; 2 Department of Cardiothoracic and Vascular Surgery, Westpfalz Klinikum, Kaiserslautern, Germany.; 3 Department of Radiology, University Hospital of Patras, Patras, Greece.; 4 Department of Pathology, University Hospital of Patras, Patras, Greece.

**Keywords:** Mediastinitis, Pulmonary Edema, Constriction, Pathologic, Arterial Occlusive Diseases/etiology, Pulmonary Veins/Pathology

## Abstract

Sclerosing mediastinitis (SM), previously named chronic fibrosing mediastinitis, is an inflammatory process that in its end-stage results to sclerosis around the mediastinal structures. SM is quite rare and has been correlated with inflammatory and autoimmune diseases, as well as malignancy. SM may either present in a mild form, with minor symptoms and a benign course or in a more aggressive form with severe pulmonary hypertension and subsequent higher morbidity and mortality. The diagnosis of SM may be difficult and quite challenging, as symptoms depend on the mediastinal structure that is mainly involved; quite often the superior vena cava. However, practically any mediastinal structure may be involved by the fibrotic process, such as the central airways, as well as the pulmonary arteries and veins, leading to obstruction or total occlusion. The latter may be impossible to undergo proper surgical excision of the lesion, and is considered to be a real challenge to the surgeon. We herein report a case of SM that presented with arterial and venous compression. The imaging appearance was that of unilateral pulmonary edema, associated with lung collapse. The case is supplemented by a non-systematic review of the relevant literature.

**Table:** 

Abbreviations, acronyms & symbols	
CRP	= C-reactive protein
CT	= Computed tomography
ESR	= Erythrocyte sedimentation rate
MAA	= Macroaggregated albumin
MRI	= Magnetic resonance imaging
PET	= Positron emission tomography
SM	= Sclerosing mediastinitis

## INTRODUCTION

Sclerosing mediastinitis (SM), formerly known as chronic fibrosing mediastinitis, represents a slowly progressive inflammatory process causing sclerosis around the mediastinal structures. It is a relatively rare condition that has been associated with various causes, such as infections, inflammatory diseases, autoimmune disorders, and malignancies^[1]^. The spectrum of the disease varies from benign with minor symptoms, to severe with pulmonary hypertension resulting in death^[2-5]^. SM is a diagnostic challenge to the surgeon, since it may involve various mediastinal structures in the fibrosing process, resulting in compressive and/or occlusive phenomena; most commonly the superior vena cava. Involvement of the pulmonary arteries^[6-9]^, the pulmonary veins^[10-15]^, or the central airways^[1,16-20]^ have also been described. Since complete surgical excision of the lesion is sometimes impossible, an alternative treatment approach may be necessary^[2,21-27]^.

We report herein a rare case of SM, causing both arterial and venous compression, resulting in unilateral pulmonary edema, which initially presented clinically as lung atelectasis. Furthermore, a non-systematic review of the relevant literature is also provided.

## CASE REPORT

**Fig. 1:**
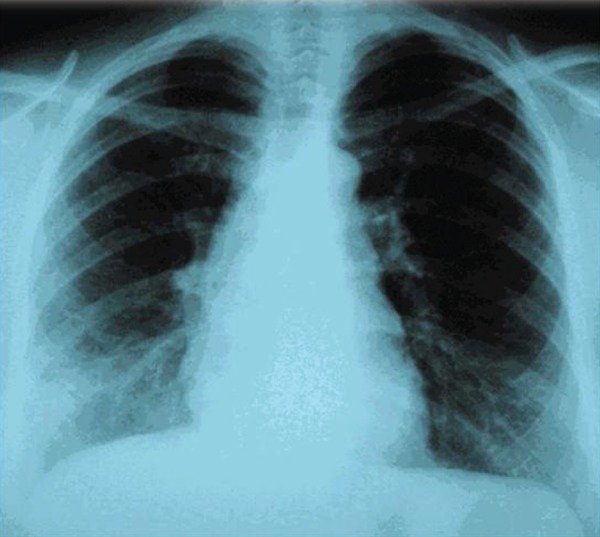
Posterior-Anterior (PA) chest X-ray. There is an ill-defined opacity in the right lower lung zone, with some degree of fine reticulation, extending to the lung periphery (Kerley B lines).

**Fig. 2:**
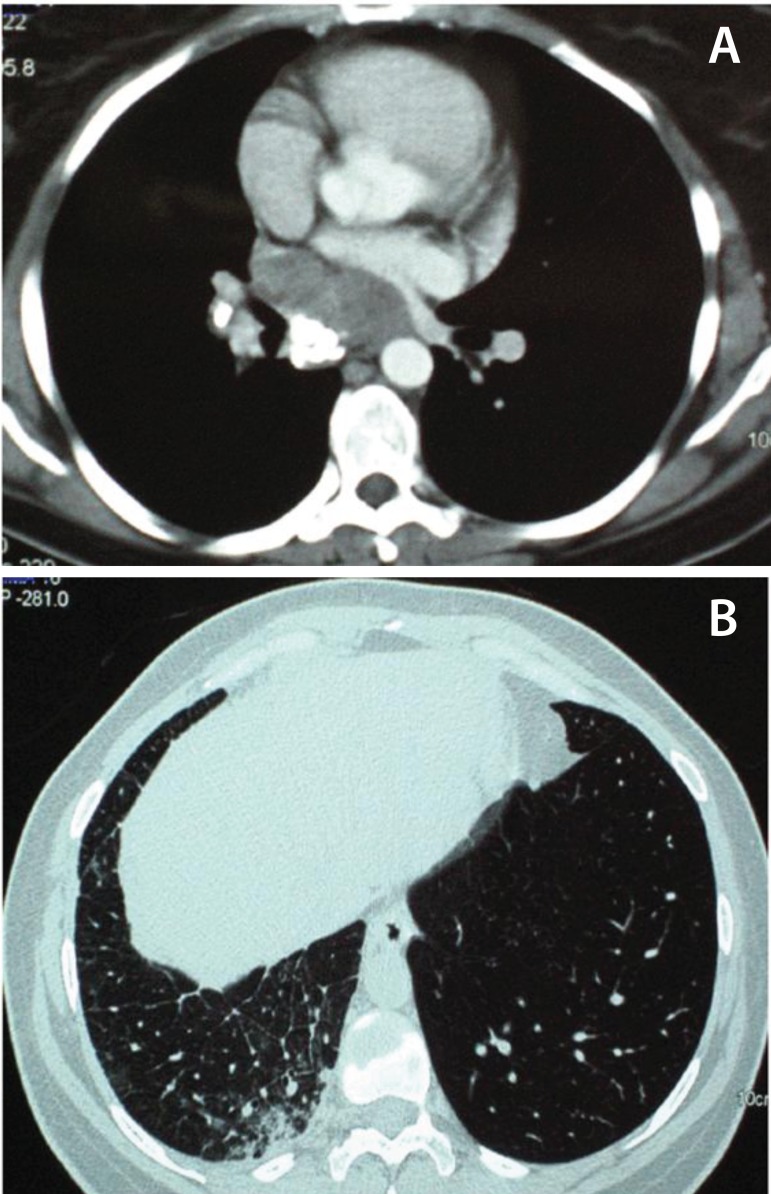
A: Axial contrast-enhanced lung CT (mediastinal windows): There is a soft tissue attenuation mass at the level of the right lower pulmonary veins. The lesion abuts the posterolateral part of the right atrium. The mass exhibits coarse calcifications in its posterior part. Note the presence of a calcified bronchopulmonary node on the right, which could be attributed to a previous granulomatous infection. B: High resolution CT scan (lung windows) at the level of the lung bases, depicting thickened interlobular septa, patchy ground glass opacities and infiltrates, consistent with regional lung congestion due to impaired pulmonary venous return.

**Fig. 3:**
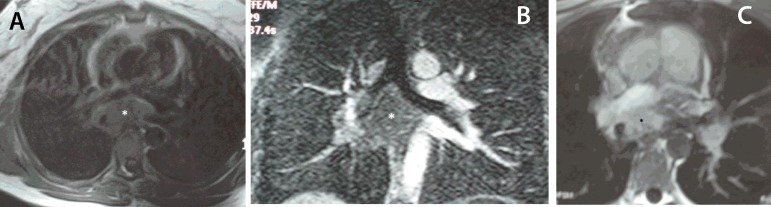
A: Axial T1-weighted image. The mass is almost isointense to the thoracic muscles (white asterisk). B: Coronal gradient-echo T2 image. The mass extends upward to the subcarinal region. The lesion exhibits a heterogeneous hypointensity (white asterisk), consistent with the presence of fibrous component. C: Axial T1-weighted image, post-gadolinium image. The mass (black asterisk) shows contrast uptake, more homogeneous in its anterior part. Note that the uptake is less homogeneous in its posterior part. Contrast uptake may represent areas with active inflammation.

A 35-year-old female presented with dyspnea on exertion, fatigue and incidents of tachycardia over the past 8 months. She also complained about intermittent, irritating cough sometimes accompanied with blood-streaked sputum. The patient reported being previously on oral contraceptives, for polycystic ovary disease, for which she was finally operated. Because of increasing shortness of breath, she underwent pulmonary workup, where the lung functional tests, including spirometry and carbon monoxide diffusing capacity, were normal. Chest X-ray showed a right lower lobe opacity (Figure 1). Transthoracic echocardiography revealed a 42 mm left atrial dilatation, accompanied with moderate tricuspid valve regurgitation, moderate pulmonary hypertension and a measured pulmonary artery systolic pressure of 42 mmHg. A chest computed tomography (CT) scan (Figure 2) revealed a solid mass, measuring 7 mm in diameter, in the lower part of the posterior mediastinum, extending 6.3 cm downwards from the level of the carina. The mass was impinging upon the posterior surface of the left atrium, the pulmonary vein orifices and was abutting the right hilum. Calcifications were evident within the lesion. Areas of ground glass opacities were noted in the right middle and lower pulmonary lobes, with thickening of the interlobular septa especially at the periphery of the lung parenchyma, indicative of pulmonary vein inflow obstruction. Subsequently a chest magnetic resonance imaging (MRI; Figure 3), confirmed the presence of the space occupying lesion, extending to the subcarinal region. The mass was compressing the right main pulmonary artery, the peripheral part of which did not exceed 7 mm in diameter; it was also in close relation to the azygos vein, the esophagus, the central part of the right mainstem bronchus and the right wall of the descending thoracic aorta. Furthermore, it compressed the posterior aspect of the left atrium, with obliteration of the pulmonary veins. Further workup with fiberoptic bronchoscopy revealed hemorrhagic mucosa with evidence of external compression and stenosis of the right lower lobe bronchus. No endobronchial mass was found and the aspirated lavage was negative for malignancy. A lung perfusion scan with 5 mCi of 99mTc-HAM (human albumin microspheres) followed, demonstrating minimal uptake (5%) of the radioactive substance from the right lung, indicative of severe hypoperfusion.

Differential diagnosis included teratoma, lymphoma, sarcoidosis, primary lung cancer, metastatic carcinoma, mediastinal sarcoma or a mediastinal desmoid tumor.

Thoracotomy was decided in order to obtain definite diagnosis and also because of the lesion location. Mediastinoscopy was technically impossible, therefore, an exploratory right lateral thoracotomy was performed. Intraoperative findings were in accordance to the preoperative MRI and CT scans. Surprisingly, tortuous, engorged vessels demonstrating a rich collateral between the diaphragm and the azygos, as well as between parietal and visceral pleura were noted. The latter indicated a slow growing mass, which was compensated by the patient. The lesion was hard on palpation, due to the high degree of fibrosis, with areas of calcification. It was tightly adhering to the esophagus, the left atrium, the pulmonary vessels and the right bronchi. Intraoperatively, multiple frozen section biopsies were obtained, as close as possible to the center of the mass. The procedure was vigorous, due to the hard texture of the lesion; the quick-frozen sections were returned negative for malignancy. Mobilization of the esophagus to prevent dysphagia was performed. Complete removal of the lesion was impossible due to its firm adherence to the surrounding vital structures.

The patient had a relatively uncomplicated postoperative course, apart from a transient episode of partial right lower lobe collapse, revealed in the chest X-ray and a pneumonitis episode, which responded well to treatment with antibiotics; she was finally discharged on the sixth postoperative day.

**Fig. 4:**
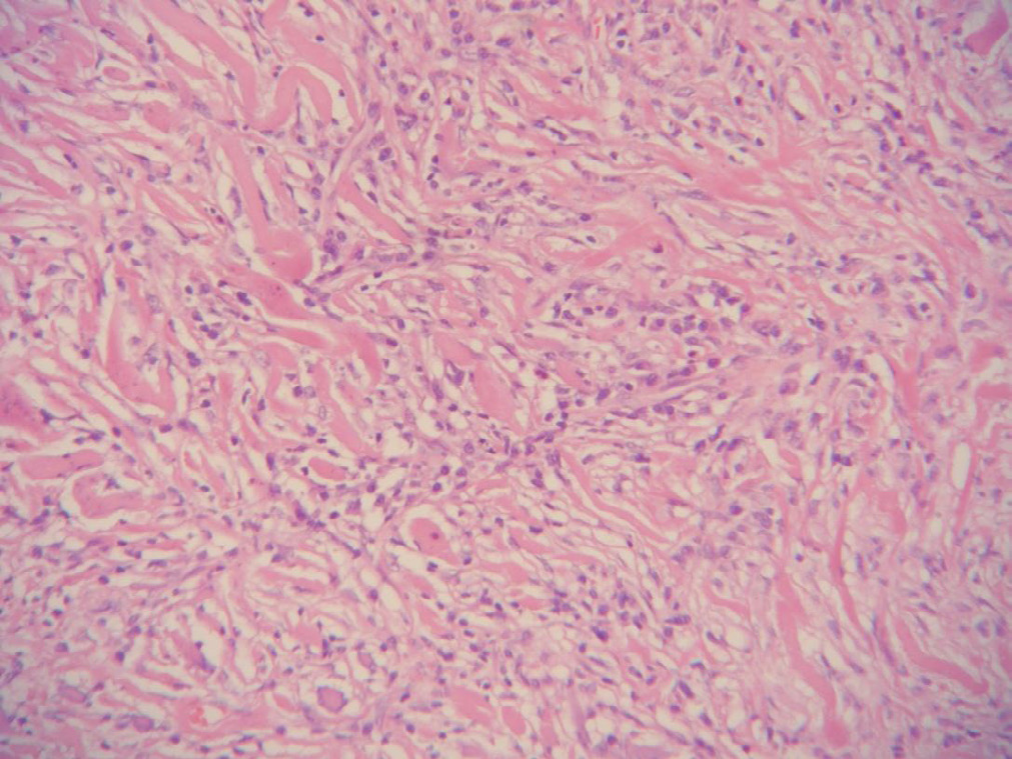
Haphazardly arranged collagen lamellae separated by an inflammatory infiltrate.

The pathologic examination revealed dense bands of collagen separated by a chronic inflammatory cell infiltrate of lymphocytes and plasma cells (Figure 4). Moreover, areas of calcification were also observed. The process extended into the pulmonary parenchyma at the level of the hilum. Stains for acid-fast bacilli and fungi were negative.

After an episode of hemoptysis, she was administered steroid treatment (methylprednisolone), based on case study reports; treatment also comprised azathioprine, calcium, vitamin D3, isoniazide and vitamin B6 (due to positive Mantoux test). The patient received an initial dose of 20 mg/d methylprednisolone, which was tapered over the next few weeks to a maintenance dose of 5 mg/d. At the beginning of the treatment, the patient demonstrated elevated inflammatory markers [erythrocyte sedimentation rate (ESR), C-reactive protein CRP)], which were significantly decreased a month later. She also experienced several side effects of steroid treatment, including central obesity, facial hair increase, acne and myopathy.

**Fig. 5:**
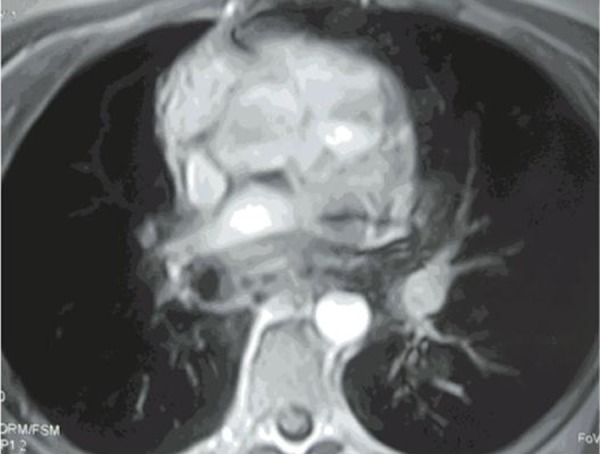
Post-treatment, follow-up, axial T1-weighted, post gadolinium image. The mass has smaller dimensions and the contrast uptake is less intense

During follow-up, an MRI scan performed two years postoperatively demonstrated 15% decrease in the dimensions of the lesion. A new MRI scan was repeated nine months after the previous one, which showed that the dimension of the lesion remained unchanged (Figure 5). A new pulmonary workup showed significant improvement in lung functional tests, compared to those two years ago. Clinically, the patient reported moderate improvement of her dyspnea. Additionally, postoperative echocardiography showed a measured pulmonary artery systolic pressure of 15 mmHg. No definite etiological factor was detected, while the patient was on follow-up. Unfortunately, the patient was lost to follow-up 4 years postoperatively.

## DISCUSSION

Fibrosing mediastinitis, also known as SM was first reported by Hunter in 1757 and later described by Hallet in 1948^[28]^. The disease affects mostly young adults between 30 and 45 years old. SM is characterized by deposition of fibrous tissue around mediastinal structures. Men are more frequently affected than women^[20,29]^. SM as well as a spectrum of other disorders, such as retroperitoneal fibrosis, sclerosing cholangitis, Riedel's thyroiditis, and orbital pseudotumor are considered "different manifestations of a single disease"^[25]^.

SM is a rare, benign condition. It occurs due to extensive proliferation of acellular collagen and fibrous tissue within the mediastinum^[30]^. It is a manifestation of an altered immunological reaction which results in diffuse dense fibrosis, which infiltrates mediastinal structures^[31]^. In the majority of patients, no etiological factor can be found and in such cases SM is considered idiopathic. Flieder et al.^[32]^ introduced the term "idiopathic fibro-inflammatory lesion of the mediastinum" to replace the term fibrosing mediastinitis.

Many cases of secondary SM have been linked to Histoplasma capsulatum infections^[29,33]^. Concerning the pathogenesis of SM, it is postulated to be a late hypersensitivity reaction to diseases such as fungal infections (aspergillosis, cryptococcosis, blastomycosis, mucormycosis), tuberculosis, syphilis, as well as, immune response to radiation, drugs, trauma, and malignancy^[7,16-19,31,34,35]^. These aforementioned factors may stimulate fibroblastic activity resulting in collagen deposition and fibrosis.

Moreover, the relationship between fibrosing mediastinitis and mediastinal granuloma is also controversial; both represent varying manifestations along the temporal spectrum of one disease process, and mediastinal granuloma is the precursor of mediastinal fibrosis^[36]^. Interestingly, Dines et al.^[37]^ reported that SM developed over a two-year period, in 34% of patients with mediastinal granuloma. The term mediastinal granuloma is commonly used in the setting of a focal mediastinal mass caused by lymph node infection due to Histoplasma capsulatum species. The lesion is typically well encapsulated, without local invasion or diffuse mediastinal fibrosis and usually without involvement of the airways, pulmonary arteries, or pulmonary veins^[33]^. Both disease entities have similar histological characteristics, while common immunological mechanisms are involved into their pathogenesis. However, their main difference lies in the areas they usually affect, as well as their extent. SM originally occurs in the mediastinum and extends to the lung parenchyma, whereas the mediastinal granuloma follows an opposite course starting from the lung parenchyma and extending to the mediastinum. In many cases both conditions are reported in the same patient, who may also suffer from other fibrosing disorders, such as retroperitoneal fibrosis^[38]^.

Prevalence and incidence information is unfortunately not available, due to lack of reliable data, probably due to the disease's unclear or multifactorial pathophysiology. Regarding the entire population who contract histoplasmosis, far less than 1% has the excessive healing response to the fungal infection, that is the basis of the disease. The incidence of idiopathic fibrosing mediastinitis is estimated to be even lower.

Sherrick et al.^[16]^ retrospectively reviewed the radiographic findings among 33 patients with fibrosing mediastinitis and identified two distinct patterns of involvement: a localized pattern noted in 82% and a diffuse pattern, in 18%. The focal pattern, manifests as a soft-tissue attenuation mass, that is frequently calcified and is usually located in the right paratracheal, in the subcarinal regions or in the hilum. It is most likely due to histoplasmosis and does not improve with steroid treatment. On the other hand, the diffuse pattern manifests as a diffusely infiltrating, non-calcified mass that involves multiple mediastinal compartments. It is not related to histoplasmosis but often occurs in the setting of other idiopathic fibrosing disorders such as retroperitoneal fibrosis. Treatment with corticosteroids may result in clinical and radiographic improvement.

Patients may be asymptomatic for a long time or present with constitutional symptoms, such as fever and weight loss. Moreover symptoms may be caused by obstruction, compression, entrapment, strangulation or invasion of any mediastinal structure, such as the central airways, superior vena cava, pulmonary veins, and pulmonary arteries^[33]^. The sites most commonly affected are the right paratracheal region, resulting in superior vena cava and azygos vein compression; the subcarinal area, with obstruction of the bronchi and the pulmonary artery laterally, while anterior extension obstructs the pulmonary vein. Obstruction of the central airways is infrequent in patients with fibrosing mediastinitis and typically manifests with cough, stridor, wheezing, dyspnea and hemoptysis or exhibits lung collapse distal to the occluded airway, as in our patient. It can also be complicated with post-obstructive pneumonia. Other structures such as the heart, the pericardium, the coronary arteries, the aorta, and the aortic branch vessels are much less frequently involved^[39]^.

Posterior mediastinal extension of the fibrosing process results in dysphagia or broncho-esophageal fistula in cases of esophageal involvement; and, finally, involvement of the right pulmonary hilum. Clinical symptoms may be vague and non-specific since patients present with cough, dyspnea, hemoptysis, dysphagia and chest pain^[29]^. The most common presentation is superior vena cava syndrome^[34]^. It can also cause pulmonary artery stenosis and pulmonary hypertension^[30,40,41]^. Patients with pulmonary venous occlusion^[10-15]^ may present with progressive or exertional dyspnea as well as with recurrent hemoptysis. This pattern of symptoms has been called the "pseudo-mitral stenosis syndrome". One of the most important causes of morbidity and mortality is longstanding pulmonary venous occlusion, leading to secondary pulmonary arterial hypertension and cor pulmonale^[42]^. Pulmonary venous occlusion may also lead to pulmonary infarction^[43]^. Pulmonary arterial stenosis or occlusion^[6-9]^ caused by SM results less frequently in pulmonary hypertension^[2-5,13,42]^. Rare cases with involvement of the coronary vessels^[44]^ or the pericardium may result in constrictive pericarditis^[7,45]^ cause by the fibrosing process. Jain et al.^[1]^ describe a case of sclerosing mediastinitis trapping the ascending aorta that was radiographically thought to be an intramural hematoma. Miyata et al.^[46]^ report a rare case of asymptomatic sclerosing mediastinitis mimicking mediastinal tumor with pleural dissemination.

Patients present with an abnormal chest X-ray, depicting a mediastinal mass usually in the right paratracheal area. Kerley B lines may be also present.

CT depicts more accurately the extent of the disease, along the middle mediastinal structures and the involvement of paratracheal, subcarinal and pulmonary hilar areas, with better demonstration of calcifications, not usually obvious on routine X-rays^[7,18,19]^. It also delineates the extent of vascular involvement and obstruction as disease progresses. Pulmonary vascular occlusion may result in pulmonary hypertension presenting as areas of diffuse lung attenuation, and thickening of the interlobular septa^[20]^, as in the present case. Intra-parenchymal bronchial dilatation and wall thickening is also known to happen in patients with chronic pulmonary embolism. This may be the result of decreased pulmonary arterial caliber due to chronic obstruction, compensated by airway dilatation^[9]^.

The role of MRI in the diagnosis of fibrosing mediastinitis is questionable. T1-weighted images show a heterogeneous, infiltrative mass of intermediate signal intensity. Its appearance on T2-weighted images is more variable^[47]^; regions of both increased and markedly decreased signal intensity are frequently seen in the same lesion. MRI with flow sensitive pulse sequences can be useful for the assessment of vascular stenoses, when intravenous contrast material is contraindicated^[48]^. MR imaging can also be useful for assessing vascular patency after percutaneous or surgical treatment^[49]^. MRI poorly depicts calcifications and CT is therefore considered the mainstay for diagnostic evaluation in fibrosing mediastinitis patients^[46]^.

Echocardiography may show an increase in the right ventricular end-diastolic dimension, as well as pulmonary and tricuspid valve insufficiency and dilatation of the main pulmonary artery, proximal to the obstruction.

Perfusion scintigraphy performed with technetium-99m-labeled macroaggregated albumin (MAA) can show focal or diffuse perfusion defects in patients with pulmonary arterial or venous obstruction^[50]^.

SM is usually divided into 2 types according to radiological findings. The focal type is the most common. It is described as a localized and calcified mass in the paratracheal or subcarinal areas of the mediastinum or in the pulmonary hilum. The diffuse type is less common and typically not calcified. It presents as a diffusely infiltrating mass, affecting various structures of the mediastinum. Moreover, other findings in the lungs are also relatively common, such as infiltrates, consolidation, and pleural effusions^[38,51,52]^.

Disease severity may be indicated using whole-body fluorine-18 fluorodeoxyglucose (F-18 FDG) positron emission tomography (PET) scanning, since marked uptake on PET is correlated with active disease. PET may assess inflammatory activity of the disease, as well as the effectiveness of immunosuppressive therapy; it can also detect other autoimmune diseases associated with fibrosing effects and can identify disease relapse^[52]^. This imaging technique can be a useful tool for patient management and follow-up, helping to evaluate the results of the treatment administered^[53]^.

Accurate diagnosis of this rare condition is usually difficult since sputum cultures are rarely indicative, skin tests are nonspecific and serological tests for fungal diseases are rarely positive. Minimally invasive methods and exploratory surgery is often needed in order to make a definitive diagnosis^[35]^.

Primary or idiopathic SM is histopathologically diagnosed when secondary causes of mediastinal fibrosis have been excluded such as histoplasma, mycobacterium, and nocardia infections, as well as Hodgkin's lymphoma, sclerosing large cell lymphoma, sarcoidosis and autoimmune disorders^[33]^.

The differential diagnosis of SM includes intrathoracic desmoid, solitary fibrous tumor, Hodgkin's lymphoma, primary mediastinal large B-cell lymphoma and desmoplastic malignant mesothelioma^[38,47]^. Lesions caused by desmoid tumors are described as more cellular and vascular. The diagnosis of solitary fibrous tumor and mesothelioma can be excluded by positive immunostains for CD34 and keratins. Inflammatory myofibroblastic tumor presents with increased ALK gene expression. Multifocal fibrosclerosing lesions present with increased IgG4+ plasma cells^[38]^. Adequacy of tissue samples is very important and diagnosing sclerosing mediastinitis in small biopsy specimens should be made with caution^[38,54,55]^. The main histological characteristics are dense bundles and sheets of hyalinized collagen, with relatively sparse inflammatory infiltrate. They are microscopic hallmarks of the disease with three different stages based on the proportion of fibrous tissue to the inflammatory component^[32]^. The evolution of the disease is variable. The main causes of death in patients suffering from SM can be summed up in infectious diseases, excessive hemoptysis and heart failure due to cor pulmonale. Prognosis mainly depends lesion location and structures affected. The prognosis is very good in patients with the focal type of SM, presenting with localized mediastinal or hilar fibrosis. On the other hand, patients, who suffer from the diffuse type, involving subcarinal or bilateral mediastinal structures, have higher mortality rates, up to 30%^[29,33,38]^.

Treatment of SM can be conservative with use of antifungal or antimycobacterial agents, when diagnosis is definite for infectious disease. Corticosteroids and immunosuppressants have also been used in the treatment of SM and similar disorders, with controversial however results^[22-27]^. Most studies demonstrate that medical treatments, including steroids have relatively poor results. However, Ikeda et al.^[22]^ reported complete resolution of the disease in a patient with fibrosing mediastinitis and sclerosing cervicitis treated only with prednisolone. Joury et al.^[21]^ describe a case of idiopathic SM, associated with occlusion of three pulmonary veins and the left main pulmonary artery, which was treated with initial high-dose steroids followed by maintenance steroid and methotrexate, with very good long-term disease control. Inoue et al.^[56]^ reported a patient with SM who had elevated serum IgG4 and increased numbers of IgG4-positive plasma cells in the mediastinal lesion, suggesting an IgG4-related immunopathologic process involved in the pathogenesis of the disease, similar to sclerosing pancreatitis. That patient responded well to steroid treatment with regression of the mediastinal mass, like those suffering from sclerosing (autoimmune) pancreatitis^[38,56]^.

Tamoxifen has been administrated by Clark et al.^[26]^ in patients with retroperitoneal fibrosis with promising results. Others used a combination of tamoxifen and steroids but patients showed recurrence after cessation of treatment^[23,27]^.

In cases of SM associated with histoplasmosis, antifungal treatment can be used. It may include traditional antifungals such as amphotericin B and itraconazole or newer agents such as voriconazole and posaconazole, which have shown promising results in experimental models^[40]^. Antifungal therapy is typically not effective in SM, however, some patients may benefit from it. Urschel et al.^[57]^ reported that six individuals treated with ketoconazole demonstrated recurrence of mediastinal disease after surgical resection.

Simple conservative treatment such as diuretics may also be considered in other cases of idiopathic forms of the disease^[58]^.

In some cases, the role of surgery may be significantly important in treating SM, since it can improve the patient's symptoms and overall status, and may also be curative in localized disease^[29,59]^. Depending on the extent of the disease surgical treatment can be performed either for complete lesion resection or only for palliation^[38]^.

Loyd et al.^[33]^ have reported that surgical procedures in 71 patients had limited benefit. Only 40% of them improved in the postoperative period, while significantly high mortality, up to 20% was observed. Mathisen and Grillo^[29]^ showed that symptomatic patients who underwent mediastinal surgery had a 22% mortality rate.

In spite of these disappointing results, surgical approach is still an important option in certain patients. Dunn et al.^[59]^ reported a case of a patient who presented with severe right pulmonary artery stenosis. They performed successful Dacron bypass grafting and the patient's heart failure resolved and the graft was patent for three years. Vascular or airway surgical reconstruction is demanded when complete surgical resection of the diseased structures is performed^[60]^. Brown et al.^[51]^ in their study reported successful surgical strategies to relieve pulmonary artery obstruction due to SM. They reported five patients who underwent surgical management including the creation of a double right ventricular outflow tract and complete reconstruction of the PA confluence or even a hybrid technique of pericardial reconstruction and stenting^[51]^. All patients showed significant reduction of right ventricular function.

The surgical approach is also crucial for diagnostic reasons. In most cases tissue samples obtained by percutaneous needle technique may be limited in size and thus insufficient to rule out malignancy; therefore, open biopsy is necessary in order to reach a definitive diagnosis. As mentioned above, neoplasms such as solitary fibrous tumors of the pleura, sclerosing non-Hodgkin lymphoma and the nodular sclerosis variant of Hodgkin's disease are neoplasms included in the differential diagnosis, and a biopsy sample is needed to exclude them^[59,60]^. Surgical treatment is usually contraindicated in cases of bilateral mediastinal involvement. Overall, surgical approaches have not been relatively promising, so far. High morbidity and mortality rates have been noticed in patients who underwent extensive surgical resections. Surgery is also rarely indicated in patients with SM associated with histoplasmosis, since fatal complications may occur due to intense mediastinal fibrosis^[29]^. If surgery is deemed necessary, it should be performed as early as possible in the disease course and only by experienced surgeons, in order to increase the possibilities of a favorable outcome^[29,41]^.

Percutaneous intravascular stenting techniques have recently become an alternative treatment option, with divergent but promising results. Stenting of major hilar structures has been used for palliation^[38]^. It is an effective treatment for central vascular obstruction, caused by SM, providing significant relief of anatomic obstruction and sustained clinical improvement^[61]^. It has also been used for the treatment of superior vena cava obstruction in cases of SM caused by histoplasmosis^[51,52]^. Smith et al.^[30]^ presented a case of an adult with pulmonary artery stenosis due to fibrosing mediastinitis induced by histoplasmosis which was successfully treated with cutting balloon angioplasty and stent placement. Doyle et al.^[62]^ showed that such stents could be applied in stenotic lesions in both pulmonary arteries and veins. However, they were unable to establish flow in all stented vessels because of in-stent restenosis. Intense fibrotic reaction, causing chronic vascular obstruction, and resulting in irreversible vascular remodeling is considered responsible for restenosis^[62]^. Restenosis limits the long-term effectiveness of percutaneous intravascular stenting techniques. It has been well described in patients with pulmonary artery stenosis due to histoplasmosis. It is believed that less pulmonary artery in-stent restenosis may occur after cutting balloon procedures^[30,63]^.

## CONCLUSION

In our patient, complete mass removal was technically not feasible, due to intense fibrosis of the lesion and its firm adherence to the neighboring vital structures. Partial removal was only possible and mobilization of the esophagus was performed in order to prevent dysphagia. According to the literature, since surgical removal is associated with high mortality rates and even though after removal of the lesion the amelioration of symptoms may not be possible, conservative treatment was decided for our patient based on the data of the preoperative exams in conjunction with the histopathological results. Postoperatively, our patient received antituberculous therapy, combined with corticosteroids and showed moderate symptomatic improvement, mainly concerning her dyspnea, and marked decrease of her pulmonary hypertension. Long-term follow-up reached four years postoperatively. No episodes of recurrent hemoptysis were observed until then. She was later lost to follow-up.

**Table:** 

Authors' roles & responsibilities	
NP	Conception or design of the work; acquisition, analysis, or interpretation of data for the work; final approval of the version to be published
VL	Conception or design of the work; acquisition, analysis, or interpretation of data for the work; final approval of the version to be published
PK	Conception or design of the work; acquisition, analysis, or interpretation of data for the work; final approval of the version to be published
PR	Conception or design of the work; acquisition, analysis, or interpretation of data for the work; final approval of the version to be published
EK	Conception or design of the work; acquisition, analysis, or interpretation of data for the work; final approval of the version to be published
DD	Conception or design of the work; acquisition, analysis, or interpretation of data for the work; final approval of the version to be published
